# Predefined Diets in Patients with Inflammatory Bowel Disease: Systematic Review and Meta-Analysis

**DOI:** 10.3390/nu13010052

**Published:** 2020-12-26

**Authors:** José M. Comeche, Ana Gutierrez-Hervás, José Tuells, Cesare Altavilla, Pablo Caballero

**Affiliations:** 1 Department of Community Nursing, Preventive Medicine and Public Health and History of Science, University of Alicante, San Vicente del Raspeig, 03690 Alicante, Spain; josemiguelcomeche@gmail.com (J.M.C.); tuells@ua.es (J.T.); eatingfaster@gmail.com (C.A.); pablo.caballero@ua.es (P.C.); 2 Department of Nursing, University of Alicante, San Vicente del Raspeig, 03690 Alicante, Spain

**Keywords:** inflammatory bowel diseases, nutrition therapy, systematic review, meta-analysis, Crohn’s disease

## Abstract

Inflammatory bowel disease (IBD) is a chronic disease mediated by the immune system and characterized by the importance of diet in pathological development. This study aims to understand how the use of predefined diets can affect the adult population diagnosed with IBD. We conducted a systematic review and meta-analysis. From the different databases (MEDLINE, Scopus, Cochrane, LILACS, CINAHL, and WOS), we found 4195 registers. After a review process, only 31 research studies were selected for qualitative synthesis and 10 were selected for meta-analysis. The variables used were Crohn’s Disease Activity Index (CDAI) for patients with Crohn’s Disease (CD) and fecal calprotectin (FC), C-Reactive Protein (CRP), and albumin (ALB) for patients with IBD. Predefined diets have been shown to have partial efficacy for the treatment of IBD and are compatible with other medical treatments. CDAI improved but with reasonable doubts due to the high heterogeneity of the data, while no differences were observed for ALB, FC, and CRP. More studies that evaluate the influence of predefined diets on IBD patients are needed due to the great variability in diets and the tools used to measure their effects.

## 1. Introduction

Inflammatory bowel disease (IBD) is characterized by chronic inflammation of the gastrointestinal tract with periods of remission or recurrence and includes both Crohn’s disease (CD) and ulcerative colitis (UC) [1].

In CD, inflammation can be present in any area of the entire gastrointestinal tract, whereas in UC, the inflammatory process affects only the colon [2].

The symptoms of this type of disease are diverse, including the appearance of diarrhea, bloody stools, abdominal pain, fatigue, weight loss, etc. [3]. The prevalence exceeds 0.3% in North America, Oceania, and many European countries, and the incidence of these types of pathologies has increased rapidly in recently industrialized countries, producing a high burden on health systems [4,5].

IBD, apart from being treated with expensive medical treatments to alleviate its activity, leads to a decrease in the patient’s quality of life that can affect the degree of disability and work productivity and is associated with more symptoms of anxiety and depression [6,7,8]. In addition, malnutrition and specific nutritional deficiencies are frequent among these types of patients, depending on the state and/or progress of the disease, and most patients impose dietary restrictions based on their own beliefs [9,10].

The etiology of IBD is unknown; in fact, it is a multifactorial disease. However, Westernised lifestyles and diets are one of the main drivers of the increasing incidence [11,12]. Diet plays an important role in the gut’s microbial composition and functioning, intestinal barrier, host immunity, and intestinal physiology [12,13].

Regarding nutritional treatment, for artificial nutrition, such as enteral and parenteral nutrition, there is evidence of efficacy [14,15], but for natural nutrition, it seems that a diet that is low in fiber, high in fat, and high in carbohydrates can lead to severe dysbiosis, while one richer in fruits, vegetables, and olive oil could prevent it. However, together with the role of certain foods such as meat, fish, and dairy products, their role in the development of the disease is controversial and uncertain in the absence of studies [16,17,18].

On the other hand, while for pediatric patients it seems that the implementation of predefined diets, such as the specific carbohydrate diet (SCD) or the Crohn’s disease exclusion diet (CDED), is positive [19], for the adult population, the studies performed to date have been inconclusive and the findings are inconsistent in addition to being mostly concerned with symptomatology of the inactive disease [20,21,22,23].

Therefore, there is a need for more and clearer evidence that allows health professionals to increase their knowledge in order to advise their patients on what type of specific dietary formulas or nutrients they will need that will allow them not only to comply with nutritional requirements but also to improve the outcome of their symptoms, with positive repercussions for a better quality of life [24,25].

The aim of this study is to understand how predefined diets, as interventions, can affect the adult population diagnosed with IBD, which will improve the dietary statements of current clinical guidelines [26,27].

## 2. Materials and Methods

To achieve this objective, a systematic review was conducted in agreement with the procedures and verification list described by PRISMA [28]. Afterwards, a meta-analysis on the more common results was conducted.

### 2.1. Systematic Review

A search of scientific works was conducted in the MEDLINE database, through the open retrieval system on the Internet such as PubMed, the Cochrane Library, Scopus, Web of Science, CINAHL, and LILACS. Studies conducted up to 10 January 2020 were compiled.

#### 2.1.1. Inclusion and Exclusion Criteria

The studies selected had to comply with the following inclusion criteria: refers to an adult population (older than 18) diagnosed with IBD; studies the effect of predefined diets within IBD; be clinical trials and observational studies; be in English, Spanish, Portuguese, French, or German.

The following articles were excluded: those that referred to the infant population, to animals, or to the use of predefined diets in a healthy adult population and those that sought the effect of specific foods or nutrients in IBD, without a clear diet designation, that were case report studies, or that were based on secondary sources.

#### 2.1.2. Search Equation

To include content linked to the intervention and predefined diets, two specific descriptors were used, such as “Nutrition Therapy” and “Diet”, and the same terms were used in the title or abstract.

For content linked to the population, we utilized the descriptor that referred to the disease, “Inflammatory bowel diseases”, and its equivalent term in the title or abstract.

Also, the filters “Humans” and “Adult” were utilized to achieve our objective.

Therefore, the main search equation designed for this study was as follows:

((“Nutrition Therapy” [Mesh] OR “Nutrition Therapy” [Title/Abstract] OR “Diet” [Mesh] OR “Diet” [Title/Abstract]) AND (“Inflammatory Bowel Diseases” [Mesh] OR “Inflammatory Bowel Disease” [Title/Abstract])) AND (Humans [Mesh] AND adult [MeSH])

The search equation was adapted to each and all of the databases described previously. The process was conducted in the period from December 2019 to January 2020.

#### 2.1.3. Selection Process

After eliminating duplicate records, the process of selection was conducted in two phases. The first consisted of reviewing the titles and abstracts of all the article records resulting from the adapted search equations and shown by the databases by using the inclusion and exclusion criteria and the objective of the study as the screening measures. The screening and selection of the records/articles were conducted independently by the two researchers, both experts in the fields of nutrition. These researchers agreed on the discrepancies found in order to define the final suitability of the records/articles found in the databases. The precision of the search was calculated based on the ratio of full-text articles selected for the review divided by the number of records found by the search equation and multiplied by one hundred.

The second phase involved the application of inclusion/exclusion criteria to the complete text of all the scientific studies selected in the first phase, thus ensuring the relevance of each one of them. In order to obtain studies that were not accessible via the Internet, we used three methods: Researchgate, the corresponding author, and interlibrary loan.

#### 2.1.4. Evaluation of the Quality of the Studies

Evaluation of the methodological quality of the included studies was performed by two independent researchers, using the CONSORT (Consolidated Standards of Reporting Trials) guide for clinical trials and STROBE (Strengthening the Reporting of Observational studies in Epidemiology) for observational studies.

These guides contain a list of 25 (CONSORT) and 22 (STROBE) essential aspects that should be described in the publication of these studies. For each selected study, one point was assigned for each item present (if not applicable, it was not scored). When an item was composed of several points, these were evaluated independently, giving the same value to each of them, and subsequently an average was made (being the final result of that item), so that in no case could it beat the score of one point per item [29,30,31].

### 2.2. Meta-Analysis

To calculate the effect size of the enteral nutrition on the variables Crohn’s Disease Activity Index (CDAI), albumin (ALB), Fecal Calprotectin (FC), and C-Reactive Protein (CRP), a meta-analysis was performed. For this, the model of fixed effects and the model of random effects were used. The results are presented as a forest plot along with the percent heterogeneity and its confidence interval at 95%, the t-value, and the heterogeneity test.

To explore the influence of each study over effect size, we used a leave-one-out method; pooled estimates were calculated omitting one study at a time. In addition, we plotted a scatterplot introduced by Baujat et al. [32]. On the x-axis, the contribution of each study to the overall heterogeneity statistic was plotted. On the y-axis, the standardized difference of the overall treatment effect with and without each study was plotted; this quantity describes the influence of each study on the overall treatment effect. Therefore, studies that fall on the top right quadrant of the Baujat plot have the most influence.

Publication bias occurs only when favorable results are published, and this could have consequences on the results of the meta-analyses if these are included. To analyze the publication bias, a nonparametric analysis was conducted, as proposed by Duval and Tweedie [33] based on the funnel-plot, by estimating and adjusting for the number and outcomes of missing studies in the meta-analysis. Another less-conservative proposal to estimate the number and outcomes of missing studies is the proposal by Copas et al. [34].

A meta-regression could not be performed due to the low number of studies. All calculations were performed within an R programming environment utilizing the packages meta version 4.15-1 [35] and metasens version 0.5–0 [36].

## 3. Results

### 3.1. Systematic Review

**Figure 1:**
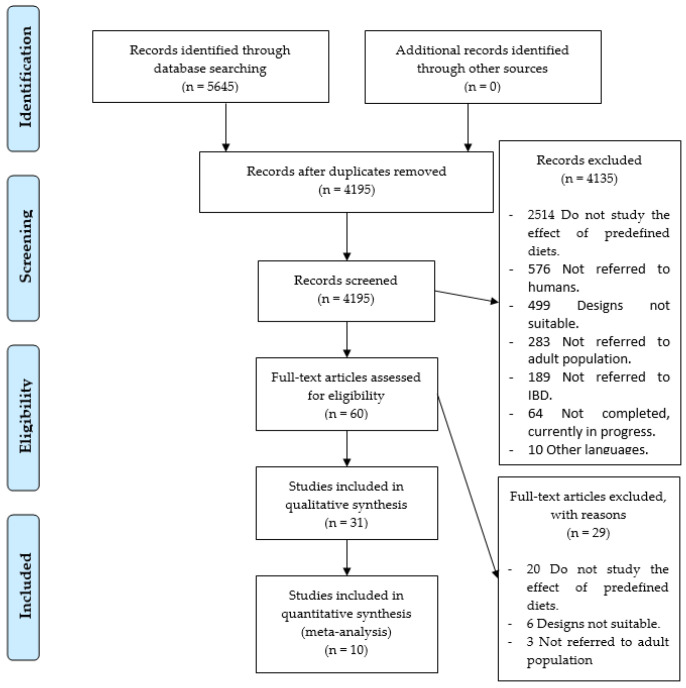
Identification and selection of studies/records in the databases.

As a result of the specific search equations used in the different databases, a total of 5645 records of scientific articles were found. A total of 1450 records were duplicated, leaving a total of 4195 records without duplication. In the first phase of the study, exactly 4135 study records were discarded, leaving 60 full-text studies to review, so that the accuracy was 1%. As shown in Figure 1, 2514 records did not study the effect of predefined diets, 576 did not refer to humans, 499 showed that the study utilized a design that was not adequate, 283 did not use an adult population, 189 did not refer to IBD, 64 were still being conducted without showing results, and 10 were written in another language other than the ones cited above.

In the second phase, 29 studies were removed: 20 because they did not investigate the effects of predefined diets, 6 due to defects in design, and 3 because the patients studied were not adults. Therefore, only 31 research studies [37,38,39,40,41,42,43,44,45,46,47,48,49,50,51,52,53,54,55,56,57,58,59,60,61,62,63,64,65,66,67] were selected, as shown in Figure 1.

As for the designs of the experimental studies included, 13 controlled and randomized clinical studies (41.9%); 3 non-randomized, controlled clinical trials (9.7%); and 3 non-randomized, non-controlled clinical trials (9.7%) were found.

Lastly, for the designs of the observational studies included, 6 cross-sectional studies (19.4%), 3 cohort studies (9.7%), 2 retrospective cohort studies (6.5%), and 1 case-control (3.2%) study were found.

**Figure 2:**
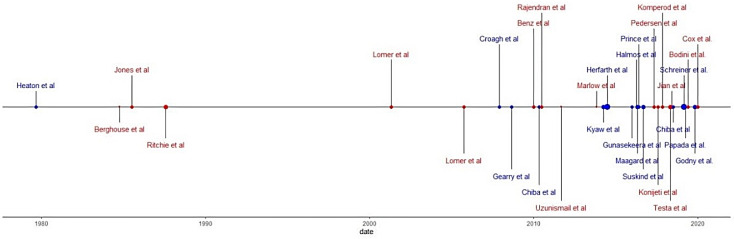
Chronological review according to type of study and sample size: red points, clinical trials; blue points, observational Studies.

In addition, 14 of the studies found showed results that specifically referred to CD, 4 studies referred to UC, and 13 studies showed results for both UC and CD under the category of IBD. Also, 8 studies mentioned the results of the disease in its active form, 3 studies reported disease outcomes of patients under surgery, 15 studies used a population with IBD in remission, and 5 studies did not indicate disease status. Figure 2 shows this information in a chronological manner.

As for the variety of predefined diets used in the studies, a total of 17 different types were found as shown in the Table 1.

The total population analyzed in the research studies found included a total of 5331 individuals with IBD: 829 diagnosed with CD and 422 with UC.

The main tools utilized by the researchers to obtain results were scores, biomarkers, and tests to measure the activity of the disease: the Crohn’s Disease Activity Index (CDAI), the Harvey–Bradshaw Index (HBI), the Van Hees index (VHI), the Modified Truelove and Witts activity index (MTWAI), the Mayo score (MS), the partial Mayo score (PMS), irritable bowel syndrome severity score system (IBS-SSS), Copenhagen IBS disease courses (CIBSC) visual analogue scales (VAS); biomarkers such as CRP, ESR, the white blood cell count (WBC), levels of albumin (ALB), pre-albumin (PA), transferrin (TRF), hemoglobin, platelet count (PL), alkaline phosphatase (ALP), etc.; and medical tests such as an ileocolonoscopy. Complementary tests were also included, such as urine, feces samples, and Bristol stool (BS) tests. Tests that measured the body’s composition were also found, such as anthropometries and bioimpedance, to obtain parameters such as body weight (BW) and body mass index (BMI). Quality of life questionnaires included the IBD Questionnaire (IBDQ), the short IBD Questionnaire (SIBDQ), the United Kingdom version of IBDQ (IBDQ-UK), and the irritable bowel syndrome quality of life questionnaire (IBS-QOL).

Table 2 schematically shows the main results found in the selected articles. Table 3 and Table 4 show the scores obtained by the studies for their methodological quality according to the CONSORT and STROBE guidelines.

### 3.2. Meta-Analysis

**Figure 3:**
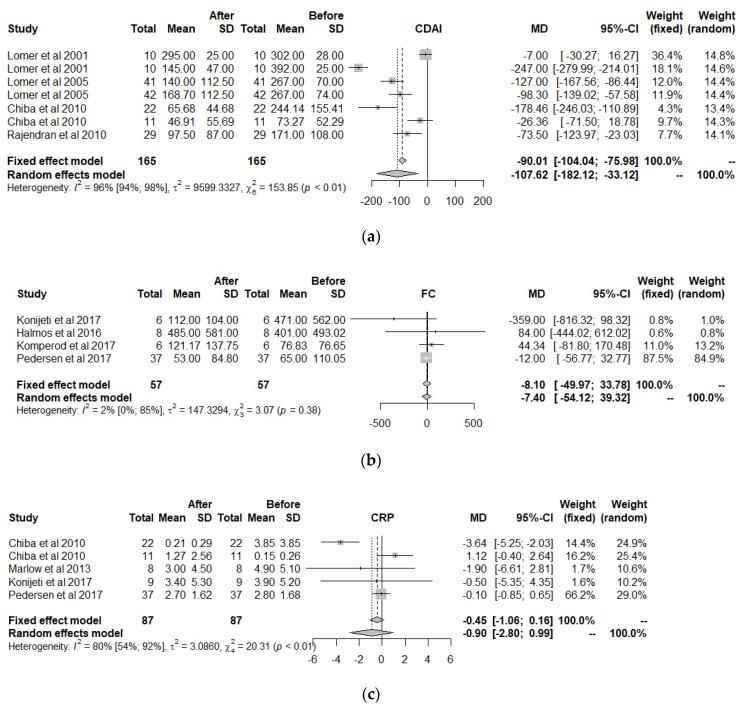

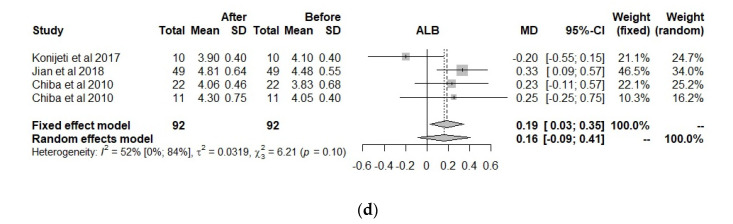
Forest plot for (**a**) Crohn’s Disease Activity Index (CDAI), (**b**) Fecal Calprotectin (FC), (**c**) C-Reactive Protein (CRP), and (**d**) Albumin (ALB).

Only 10 clinical trials had the common quality and variables needed to be used in the meta-analysis. These 10 trials worked with a total of 13 groups. The final size of the sample was comprised of 558 observed moments for 279 individuals, all with IBD, to which a predefined diet had been prescribed. The common variables were the CDAI, FC, CRP, and ALB (Figure 3).

For the CDAI, which is an index of disease activity used in patients with CD [68], the effects were positive when comparing the situation at the start and at the end of treatment with a predefined diet, independently if the situation with fixed effects (less probable) or random effects (more acceptable) was considered. However, for FC, CRP, and albumin, the use of a predefined diet was not significant. As for heterogeneity, the CDAI obtained very high values, which indicates a lack of studies, and to a lesser degree, the heterogeneity is shown in CRP (80%), while for albumin (52%) and the FC (2%), the heterogeneity is not significantly high. This could indicate the high influence of some studies or the lack of them.

The influence of each study on the results of the meta-analysis is shown in Table 5, considering a model of random effects. For CDAI, the study from 2001 by Lomer et al. was the most influential; however, it is not sufficient for eliminating the high heterogeneity, and this corroborates the need for more studies or other covariables that could explain this heterogeneity. However, there are not enough studies to perform an analysis of moderators or meta-regression.

**Figure 4:**
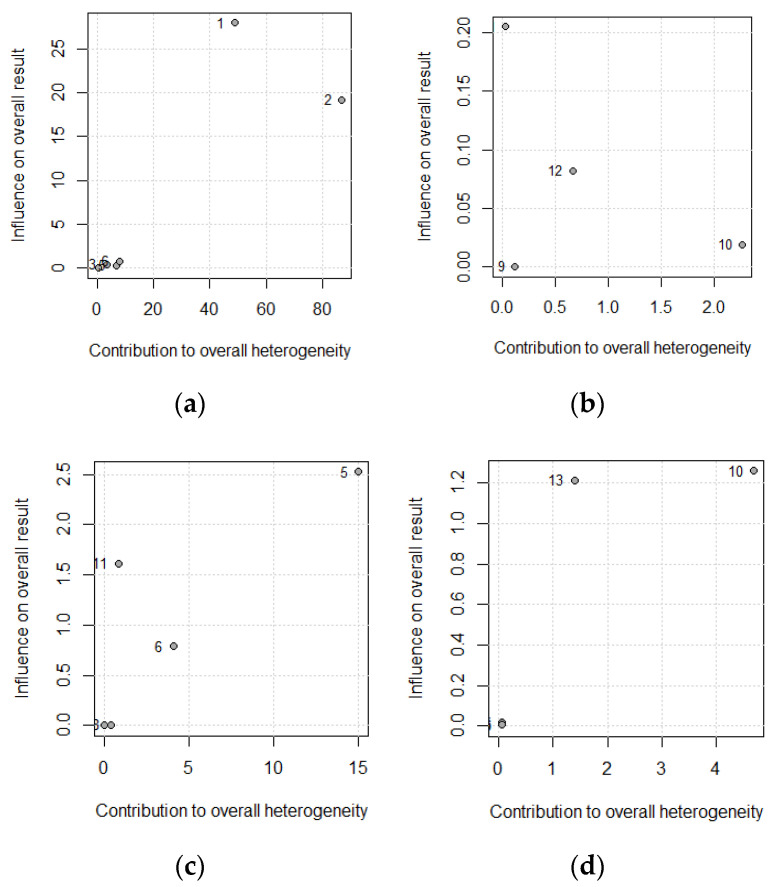
Baujat plot for (**a**) Crohn´s disease activity index, (**b**) fecal calprotectin, (**c**) C-reactive protein, and (**d**) albumin: the numbers in the plot correspond to the classification shown in Table 5.

FC and ALB are not very heterogeneous, with 2.3% and 51.7%, respectively. By removing the study by Konijeti et al., 2017, the heterogeneity decreased in both cases, meaning it was the most discrepant study. Regarding CRP, the study by Chiba 2010 introduced heterogeneity at a month and a half, but at 24 months, it did not contribute. If heterogeneity is not eliminated, it can be deduced that more studies are needed. These results are reflected in the Baujat Plots (Figure 4); the studies by Konijeti et al., 2017 (ID 5) and Chiba 2010 (ID 10) introduce the most instability in the results of CRP and ALB.

## 4. Discussion

Our systematic review included a total of 31 studies, which compiled information from 5331 individuals with IBD and who had an intervention with different predefined diets. All the studies had a broad reach, and within the diverse effects found, CDAI, FC, CRP, and ALB were the most common, allowing us to conduct a meta-analysis to arrive at more complete conclusions.

The main premise of these types of diets was based on the reduction of some types of pro-inflammatory foods and the increase of others, which are believed to promote a favorable intestinal microbiota [69]. In combination with the high prevalence of malnutrition, the importance of diets that can modify the intestinal barrier and host immunity must be increased [70,71]. In fact, although we did not observe an amelioration in terms of ALB, CRP, and CF levels, an improvement in CDAI levels was observed through interventions with predefined diets, more specifically of microparticles diet, semi-vegetarian diet, and immunoglobulin exclusion diet, in patients with CD.

The low FODMAP diet (LFD) reduces fermented oligosaccharides, disaccharides, monosaccharides, and polyols because they are poorly absorbed in the small intestine and are fermented by bacteria in the colon, triggering intestinal discomfort and gas in sensitive individuals [72,73,74]. This diet has been used mainly with patients with irritable bowel syndrome; however, it has been transferred to patients with IBD due to the similarity of functional gut symptoms such as bloating, abdominal pain, wind, and diarrhea [44,74]. As for the results obtained in our systematic review, most of the individuals improved their symptoms of the disease [44,45,59,65]. This coincides with other studies, in which an improvement was reported due to the use of an LFD for the treatment of gastrointestinal symptoms [23,75]. Furthermore, according to Pedersen et al., Testa et al., Bodini et al., and Cox et al., the LFD reported a better quality of life, although it was measured with different questionnaires [51,52,56,59]. Results of good adherence to this type of diet have also been reported [44,52,59,64], but in terms of disease activity, the results have been controversial; while for some authors no improvements were found for biomarkers or indices such as CRP, FC, HBI, or IBS-SSS, others did obtain improvements [44,51,52,56,59].

All of this, together with the concern of several authors who expressed the possibility that this type of diet may alter the microbiome by increasing the colonic pH, thereby allowing enteropathogenic colonization and causing an increase in dysbiosis [69,76,77], indicate that the use of supplementation should be considered to avoid deficiencies that could be caused by an LFD for long periods of time. Furthermore, it is of great importance that it be considered in the “induction” phase of prescription of diet modification, and if patients do not respond to the modification, the FODMAP restriction should be discontinued [76], as it can compromise the nutritional status of the patient and, to some extent, can affect intestinal inflammation [77].

The Specific Carbohydrate Diet (SCD) is based on the hypothesis that IBD patients have a dysfunction of disaccharidases, which are necessary to digest and absorb disaccharides and amylopectin. Therefore, high amounts of these compounds could cause an overgrowth of bacteria and intestinal lesions which can increase the intestinal permeability, and this is why this type of diet allows foods with carbohydrates that consist only of monosaccharides and excludes disaccharides and most polysaccharides [20]. An improvement in the symptomatology and an increase in clinical remissions are the most important results reported by Suskind et al. [56].

Both SCD and LFD have the potential to contribute to vitamin D deficiency. Therefore, their follow-up and clinical evaluation is very important due to the association of this deficiency with an increased risk of surgery and hospitalization [78,79,80,81].

The Immunoglobulin Exclusion Diet (IGED) is a dietary strategy associated with the identification of foods that cause a certain degree of intolerance, meaning an IgG-mediated reaction that acts as a delayed-type hypersensitivity response to antigen exposure, all of which result in excessive protective immune responses that could lead to increased disease activity [82,83,84]. The researchers Rajendran et al., Gunasekeera et al., and Uzunismail obtained improvements in the activity of the pathology through various tools. However, contradictory results were found for symptomatology, quality of life, and certain biochemical parameters such as CRP and ALB [39,40,48,54,67].

Several authors state that vegetarian dietary patterns are associated with a decrease in serum CRP, fibrinogen, and total leukocyte concentrations [85]. This coincides with the results obtained by Chiba et al., in which an improvement in the CRP, symptoms, and certain laboratory data could be observed [46,60]. However, it can cause an increase in posttraumatic stress and poorer mental health [66].

With respect to the Mediterranean diet (MED), characterized by the consumption of important sources of fiber (cereals, legumes, vegetables, fruits, and nuts) and with a high content of chemical compounds with antioxidant properties such as flavonoids, phytosterols, vitamins, terpenes, and polyphenols [78,86], we have obtained positive results with quality of life, HBI, FC, and cholesterol [41,58,61]. Currently, there is some controversy regarding the role of this diet in IBD, as several authors indicated that a healthy diet pattern, which includes the MED, is associated with significant reductions in inflammation-related CRP [87], and other researchers concluded that this type of diet does not have significant effects on inflammatory substances [88].

Also, there is the gluten-free diet (GFD), which eliminates the gliadin protein located in wheat, barley, rye, and other grains. This diet has been traditionally used for patients with celiac disease and more recently in people with sensitivity to non-celiac gluten [89]. However, the nutrient responsible for improvement is controversial, since these cereals have more than one possible symptom inducer such as gluten, fructans, trypsin amylase inhibitors, and lectins [90,91,92]. The results from our systematic review are controversial. On the one hand, the use of this type of diet improved the symptoms of pathology; however, it could also lead to an increase in anxiety and depression, possibly due to the difficulty of adherence [50,66]. These findings coincide with the results from some authors, who state that GFD, despite the existence of data indicating low adherence, suggests a potential benefit and great utility in the management of IBD [69,79].

Despite being the first systematic review that deals with the general effects of predefined diets on adult patients with IBD, this article is not exempt from limitations. It is possible that the CONSORT questionnaire was not the best for evaluating the Non-randomized controlled clinical trials (NRCCT) and Uncontrolled and non-randomized clinical trial (UNRCT) reviewed; however, we tried to avoid this limitation by adjusting the items of this tool to the type of study, as no questionnaire was found that evaluated the Randomized controlled clinical trial (RCCT), the NRCCT, and the UNRCT [30,93]. Also, some studies were somewhat old, which could have reduced the score of this tool on the methodological quality due to the lack of standard criteria at the time the clinical trials were conducted. The UC and CD data were combined to perform a meta-analysis for the variables CDAI, FC, CRP, and ALB due to the low number of studies that separated these diseases to elaborate on their results and the great variability, not only of the tools used but also of the unit of measurement employed. However, these clinical entities have different clinical courses. The results derived from this work could help in clinical practice to help health professionals, through the creation of a guide oriented towards evaluating the addition of predefined diets within the set of medical therapies for an adult patient diagnosed with IBD. Both clinical trials and observational studies have been used within this systematic review, a parameter that has allowed us to have a more global view of the effect of intervention.

As future lines of research, the use of other types of predefined diets should be considered, which have been observed to show positive results in such patients and for which little evidence is found [37,38,41,42,49,50,53,60,62,63].

## 5. Conclusions

Predefined diets have been shown to have partial efficacy for the treatment of IBD and are compatible with other medical treatments. CDAI improved in patients with CD but with reasonable doubts due to the high heterogeneity of the data, while no differences were observed for ALB, FC, and CRP. LFD, IGED, MED, GFD, and vegetarian diets are the most studied and beneficial dietary interventions for these patients. However, there was a great variability in the diets and tools used to measure their interventions. In addition, the mechanisms of action of the food or nutrients responsible for the improvement are unknown. Thus, more studies that evaluate the influence of predefined diets on IBD patients are needed.

## Figures and Tables

**Table 1:** Predefined diets in systematic review studies.

Number of Studies	Type of Predefined Diet	Number of Studies	Type of Predefined Diet
9	Low fermentable oligosaccharides, disaccharides, monosaccharides and polyols diet (LFD)	1	Dietary modification framework for UC (DMF-UC)
5	Immunoglobulin exclusion diet (IGED)	1	Specific food intolerance diet (SFID)
4	Unrefined carbohydrate fiber rich diet (UCFR)	1	Typical Australin diet (TAD)
2	Microparticles diet (MD)		
2	Mediterranean diet (MED)	1	Specific carbohydrate diet (SCD)
2	Gluten-free diet (GFD)	1	Komperod elimination diet (KED)
2	Refined carbohydrate diet (RCD)	1	Autoimmune protocol diet (AIPD)
1	Semi-vegetarian diet (SVD)	1	Plant-based diet (PBD)
1	Mediterranean-inspired anti-inflammatory diet (MIAID)	1	Vegetarian diet (VD)

**Table 2:** Main results of the systematic review.

Author	Study	n/Age	Disease	P/m	CC	Treatment	Variables	Main Results
Heaton et al., 1979 [37]	CCS	64/36 M 31 F 33	CD UCFR/ND 32/32	52	GBR	UCFR and ND	Admission to hospital, IOP, EIMs	The number of hospital admissions required in UCFR patients was 11 compared with 34 in the ND (*p* < 0.01). UCFR patients spent a total of 111 days in hospitals compared with 533 days for ND (*p* < 0.005). Intestinal operations were performed on only one UCFR patient but on five ND.
Berghouse et al., 1984 [38]	RCCT	10/44 M 6 F 4	SUR IBD CD/UC 5/5	0.5	GBR	UCRF and RCD	7-FR, chemical analysis of the ileostomy fluid	Compliance with the dietary advice was good. A dietary assessment was possible in 7/10 patients. The amount of ileostomy effluent was significantly greater on the UCRF both in terms of wet weight (238 ± 89 g vs. 162 ± 79 g) and dry weight (23.6 ± 6.8 g vs. 14−9 ± 6−6 g).
Jones et al., 1985 [39]	RCCT	20/30.73 M 18 F 2	ACT CD EG/CG 10/10	6	GBR	EG: SFID CG: UCFR	CDAI, ESR, length of remission, orosomucoid	At 6 months, 7 patients on an SFID remained well (*p* < 0.05, Fisher´s exact test). The mean time to relapse was 1.38 ± 1.74 (SD) months in the UCFR group and 2.75 ± 1.98 months in the SFID group. For patients on the SFID who remained in remission for 6 months, the ESR (mean ± SD) dropped from 39.7 ± 21.7 mm/h before the trial to 16.2 ± 12.5 (*p* < 0.05) at 6 months and their orosomucoid concentrations dropped from 232.5 ± 68.2% before the trial to 140 ± 41.5% (*p* < 0.05) at 6 months.
Ritchie et al., 1987 [40]	RCCT	352/35.4 M 130 F 222	REM CD RCD/UCFR 162/190	24	GBR	UCFR and RCD	RC, clinical deterioration, 7-FR, BW	178 patients completed the trial. The cumulative proportion of patients remaining in the trial without deterioration of the disease was 64% in RCD and 59% in UCFR. The clinical score, stool count, and BW showed no significant changes with either diet.
Lomer et al., 2001 [41]	RCCT	20/36.2 M 3 F 17	ACT CD LMD/NMD 10/10	4	GBR	LMD and NMD	CDAI, HEMA, CA, and ALB	Despite a higher CDAI at month 0 in the LMD group, compared to the NMD group, there was a significantly lower CDAI in the LMD group by month 4 (145 ± 47 vs. 295 ± 25). All patients had normal CA levels, corrected for ALB, and there were no significant changes in nutritional status in either group.
Lomer et al., 2005 [42]	RCCT	83/36 M 40 F 43	ACT-CD LCLM/LCNM/ NCLM/NCNM 22/21/ 20/20	4	GBR	LCLM, LCNM, NCLM and NCNM	CDAI, VHI, IBDQ, ESR, CRP, FC, IP	No differences were found between the low and normal calcium groups for CDAI, VHI, IBDQ, CRP, ESR, FC, or IP, neither between the low and normal dietary calcium groups nor between the low and normal microparticle groups.
Croagh et al., 2007 [43]	CS and RCS	15/43.6 M 6 F 9	SUR IBD CS/ RCS 8/7	1.5	AUS	LFD	FL, SF, DH, adherence to diet	In the RCS, adherence was good in 5/7 patients, while in the CS, it was 3/8; 5/7 studied retrospectively improved stool frequency (from median 8 to 4 per day; *p* < 0.05). In CS, only 5 patients were evaluable for the effect of diet on SF, with no change seen. For the 7 patients without pouchitis, median daily SF fell from 8 to 4 (*p* < 0.001).
Gearry et al., 2008 [44]	CSS	72/48 M 33 F 39	REM CD/UC 52/20	3	AUS	LFD	Adherence to diet, change of gastrointestinal symptoms	Overall abdominal symptoms, abdominal pain, bloating, wind, and diarrhea improved in patients with CD and UC (*p* < 0.02 for all), but constipation did not. The median response for LFD implementation was 3/10 “easy” (SD 2.9, range 0–10, interquartile range 0.25–5).
Benz et al., 2010 [45]	RCCT	40/41 M 16 F 24	ACT CD IGED/CG 20/20	1.5	GER	IGED and CG (Sham diet)	GISD, SF. and general well-being	An average reduction in the total weekly score of 6.5 points was estimated for the IGED group compared with the CG (95% CI: −0.6, 13.6 points). The estimated effect seems to have a clinically relevant effect but is not significant (*p* = 0.07). The daily SF significantly decreased by 11% during an IGED compared with CG.
Chiba et al., 2010 [46]	UNRCT CS	22/26.5 M 14 F 8	ACT CD	UNRCT 1.5 CS 24	JPN	SVD	CDAI, CRP, BMI, ALB, CHOL, CHE, HEM, morphological studies, FFQ	Among the 16 (73%) patients who continued with the SVD, 15 maintained remission and one relapsed; the remission rate was 100% at 1 year and 92% at 2 years. The cumulative relapse rate at 2 years was significantly lower in the SVD group than in the omnivorous group. The concentration of CRP was normal at the final visit in more than half of the patients in remission on an SVD.
Rajendran et al., 2010 [47]	UNRCT	29/39.3 M 18 F 2	REM CD	1	GBR	IGED	MCDAI, ESR, CRP, ALB	The mean mCDAI score on entry to the trial was 171 ± 108, and after IGED, the mCDAI decreased to 97.5 ± 87 (*p* < 0.05). The general “well-being” rating improved from 0.88 to 0.63 (*p* < 0.05). The mean ESR fell from 22.3 ± 16.0 to 17.1 ± 15.2 (*p* = 0.032). CRP and ALB levels did not change significantly.
Uzunismail et al., 2011 [48]	NRCCT	8/40 M 1 F 7	REM CD	0.5	TUR	IGED and modifications IGED (provocation periods)	FC, CRP, ESR, WBC, PL, HBI, CDAI	The mean CDAI score before the provocation was 63 ± 29.9 and increased statistically significant to 99.75 ± 46.1 (46–183) during provocation. The HBI score, WBC, CRP, and WBC also increased significantly.
Marlow et al., 2013 [49]	UNRCT	8/45,4 M 2 F 6	ACT CD	1.5	NZL	MIAID	CRP, DNA damage, and microbiota	Subjects self-reported good adherence to diet; cholesterol levels were reduced by 20%. CRP and micronuclei numbers showed a trend of reduction after the 6-week diet; however, this was not significant.
Herfarth et al., 2014 [50]	CSS	1647/46.35 M 429 F 1218	REM IBD GFD/Non-GFD 314/1333	14	USA	GFD	GFDQ	In the GFD group, 206 (65.6%) reported that they experienced an improvement of at least 1 specific clinical symptom, which has been associated with gluten exposure; 38.3% also recounted fewer and less severe flares while being on a GFD, and 23.6% stated that they required fewer medications to control the disease. Excellent adherence was associated with significant improvement of fatigue.
Kyaw et al., 2014 [51]	RCCT	112/ < 65 M 62 F 50	UC DMF-UC/ND 61/51	6	GBR	DMF-UC and ND	IBDQ-UK, SCCAI, FFQ	The mean difference between SCCAI at week 0 and week 24 was a reduction by 1.304 (*p* = 0.0108) in the DMF-UC group. For the ND group, there was an increase in the mean differences between SCCAI at week 0 and week 32 of 0.875 (*p* = 0.0249). There were no significant differences in the IBDQ score; 69% of patients in the DMF-UC group found the dietary advice significantly or moderately helpful.
Gunasekeera et al., 2016 [52]	RCCT	76/39 M 32 F 44	REM CD IGED/CG 39/37	1	GBR	IGED and CG	SIBDQ, CDAI, HBI, CRP, FC	There was a 3.05 (0.01–6.11), *p* < 0.05, improvement in SIBDQ and 41 (10.4–71.5) in CDAI, *p* = 0.009. There was no significant difference in FC and CRP levels.
Halmos et al., 2016 [53]	RCCT	9/35 M 3 F 6	REM CD	0.75	AUS	LFD, TAD and ND	FM, fecal pH, FSCFA, VAS (symptoms), FWC, FC	FC was also similar across the three phases of the study, but in 3 subjects, it was consistently >150 μg/g. Dietary adherence during the interventional diets was good. The severity of overall gastrointestinal symptoms was significantly less on the last 14 days of the LFD at mean 13.5 mm compared with the last 14 days of the TAD at 24.8 mm
Maagard et al., 2016 [54]	CSS	180/43 M 33 F 147	IBD:49 IBS:131	16	DEN	LFD	VAS, FARS, assess satisfaction with LFD, BS, CIBSC, IBS-SSS, IBS-QOL, SIBDQ	The patients experiencing full effectiveness were greater in the IBD group than in the IBS group (42% vs. 29%, *p* = 0.08). At follow-up, the median IBS-QoL score was 75 (range: 37–145) for the IBS group and 63 (range: 36–126) for the IBD group. For the IBD patients only, the median SIBDQ score was 55; 32% of the IBS group and 37% of the IBD group were on the diet for less than 3 months, while 47% and 50%, respectively, stayed on the diet until follow-up. The overall median IBS-SSS score at follow-up was 211 (range: 16–487).
Prince et al., 2016 [55]	RCS	88/40 M 26 F 62	REM IBD	1.5	GBR	LFD	SRFGS, GRRS, BS	There was a significant increase in the proportion with satisfactory relief of their FGS following a LFD. Individual symptom severity scores decreased following LFD with the greatest reductions in scores observed for bloating and flatulence, followed by abdominal pain and lethargy. More patients reported normal consistency and normal-frequency stools following the LFD (*p* < 0.05)
Suskind et al., 2016 [56]	CSS	417/34.9 M 125 F 292	IBD	12	USA	SCD	Online survey: Disease complications, healthcare management, clinical questions (diagnosis, extent, duration, etc)	The reason for individuals starting the SCD was for avoidance of medication (49%), incomplete improvement with medication (28%), no improvement with medication (9%), and/or side effects or allergies to medication (19%). Overall, symptoms such as abdominal pain, limitations in activities, diarrhea, blood in the stool, and weight loss decreased over time; 4% reported clinical remission prior to the SCD, while 33% reported remission at 2 months after initiation of the SCD, and 42% reported both at 6 and 12 months
Komperod et al., 2017 [57]	NRCCT	12/38.41 M 4 F 8	REM CD	0.5	NOR	KED and ND	GISD, VAS (symptom intensity), FC	A significant decline in symptom intensity was consistently seen across all seven symptoms when we compared symptoms at week 2 of the ND to symptoms at week 2 of the KED.
Konijeti et al., 2017 [58]	UNRCT	15/44 M 4 F 11	ACT CD/UC 9/6	2.75	USA	AIPD	Clinical remission, complications, HBI, PMS, SF, RB, PGA, CRP, FC, ALB, SIBDQ	Clinical remission was achieved at week 6 by 11/15 (73%) study participants (6 CD and 5 UC). Mean total SIBDQ scores significantly improved from 46.5 (SD 12.5) at baseline to 53.3 (SD 10.9) at week 6 and 60.5 (SD 4.8) at week 11. From week 0 to weeks 6 and 11, mean PMS significantly improved from 5.8 (SD 1.2) to 1.2 (SD 2.0) and 1.0 (SD 2.0) for UC and mean HBI significantly improved from 7 (SD 1.5) to 3.6 (SD 2.1) and 3.4 (SD 2.6) for CD. CRP and FC did not significantly change during the study
Pedersen et al., 2017 [59]	RCCT	89/40 M 22 F 67	REM IBD LFD/ND 44/45	1.5	DEN	LFD and ND	IBS-SSS, SCCAI, HBI, FC, CRP, SIBDQ, IBS-QOL, FFQ	At 6 weeks, a significantly lower IBS-SSS score was observed in the LFD group (median IBS-SSS 115, IQR 33–169) as compared to the ND group (median IBS-SSS 170, IQR 91–288), *p* = 0.02. At week 6, a statistically significant improvement in SIBDQ was observed in those on a LFD (median 60, IQR 51–65) when compared to those on a ND (median 50, IQR 39–60). No significant differences were found between the LFD and ND groups with regards to FC and CRP change.
Chiba et al., 2018 [60]	CS	60/39 M 35 F 25	REM UC IEC/RC 29/31	42	JPN	PBD	PBDS, relapses, and remissions (improvement)	Of 57 cases, 8 (4/28 IEC and 4/29 RC) relapsed during the follow-up period. Cumulative relapse rates at 1, 2, 3, 4, and 5 years were 2%, 4%, 7%, 19%, and 19%, respectively. Mean time to relapse was 7 years 3 months. There were no differences between groups. Most patients (77%) experienced some improvement. The short- and long-term PBD scores after hospitalization were higher than baseline PBD scores.
Jian et al., 2018 [61]	RCCT	97/38.5 M 50 F 47	REM UC IGED/ CG 49/48	6	CHN	IGED and ND	MS, EIMs, BMI, ALB, TRF, PA, IBDQ, MF	MS in the ND group was significantly higher than that in the IGED group (3.52 ± 1.15 vs. 2.41 ± 0.89) and the endoscopic appearance tended to be better in the IGED group at 6 months. After dietary intervention, BMI and ALB were significantly higher in the IGED group than in the ND group (23.88 ± 3.31 vs. 21.50 ± 6.24 kg/m^2^, respectively, 48.05 ± 6.39 vs. 45.72 ± 5.48 g/L). There were no significant differences in PA, TRF, and IBDQ between the groups.
Testa et al., 2018 [62]	NRCCT	127/34.5 M 57 F 70	REM IBD IBS/IBD/CLD 56/30/41	3	ITA	LFD	IBS-SSS, SF-36, 7-FR	In the IBD population, the average IBS-SSS scores were 207 ± 88 SD at T0, 139 ± 50 SD at T1, and 73 ± 45 SD at T3 (*p* < 0.001), demonstrating a good response to LFD. In most of the SF-36 domains, there was a significant improvement from T0 to T3 for all groups; 8/127 (6.3%) patients revealed poor adherence.
Bodini et al., 2019 [63]	RCCT	55/46 M 24 F 31	REM IBD LFD/ND 26/29	1.5	ITA	LFD and ND	CRP, FC, PMS, HBI, IBDQ	After 6 weeks of treatment in the LFD group, in patients with CD, median HBI significantly decreased. A statistically significant decrease was observed in median calprotectin values. A significant increase in median IBDQ was detected. However, there were no significant differences between groups for any parameter.
Godny et al., 2019 [64]	CS	153/46 M 72 F 81	SUR UC	36	ISR	MED	FFQ, MedDiet, PDAI, CRP, FC PGA	Patients with an inactive disease tended to have a higher MED score compared to those with an active disease, but this difference did not achieve statistical significance (4.7 ± 1.8 vs. 4.3 ± 1.7). The MED score was associated with lower odds for elevated FC (adjusted OR = 0.74 [95% CI 0.56–0.99], *p* < 0.05). Patients who had highly adhered to MED (MED score ≥ 5) had lower rates of pouchitis than patients with low adherence to the MED (26% vs. 45.4%, log rank test, *p* = 0.17).
Papada et al., 2019 [65]	CSS	86/39.45 M 45 F 41	CD ACT-CD: 41 REM-CD: 45	6	GRE	MED	MedDiet, HBI, IBDQ, BW, BMI FE, HDL, LDL, TG, LDH, SGOT, SGPT, γ-GT, ALP, CRP, IL-6, and IL-10	Adherence was higher in REM-CD than ACT-CD (26.8 ± 5.0 vs. 30.2 ± 5.8, *p* = 0.005). Protein intake (*p* = 0.015) and vitamin C (*p* = 0.003) levels were individually higher in the REM-CD. In the regression models adjusted for age, sex, BMI, and smoking, HBI showed a highly significant negative linear association with the MedDiet score and IBDQ showed a positive linear association with the MedDiet score.
Schreiner et al., 2019 [66]	CSS	1313/NI M NI F NI	IBD GFD/VD 57/52	VD/GFD 193/33.5	SUI	GFD, VD and ND	DQ, clinical characteristics, SF-36	The authors did not find significant differences in either GFD or VD patient disease activities based on CDAI and MTWAI. VD patients had higher scores on the posttraumatic stress diagnostic scale and poorer mental health. A GFD was associated with lower scores in the physical and mental component survey (SF-36) and higher anxiety and depression scores.
Cox et al., 2020 [67]	RCCT	52/36.5 M 23 F 29	REM IBD LFD/ED 27/25	1	GBR	LFD and ED	IBS-SSS, GSRS, IBDQ-UK, SF, BS, HBI, PMS, 7-FC, CRP, FC, FM, FSCFA	There were 6 adverse events during the trial. There were no statistically significant differences between groups in total IBS-SSS score, HBI, PMS, FC, and CRP. The severity of flatulence, bloating, and SF was significantly lower during LFD compared with ED. Total IBDQ-UK was significantly greater following LFD (81.9, SEM 1.2) than ED (78.3, SEM 1.2). There was good adherence for both diets.

UNRCT: Uncontrolled and non-randomized clinical trial. NRCCT: Non-randomized controlled clinical trials. RCCT: Randomized controlled clinical trials. P/m: Period (months). NI: Not indicated. IBD: Inflammatory Bowel Disease. ACT: Active disease. REM: Disease in remission. SFID: Specific food intolerance diet. UCFR: Unrefined carbohydrate fiber rich diet. CDAI: Crohn’s Disease Activity Index. ESR: Erythrocyte sedimentation rate. F: Female. M: Male. MIAID: Mediterranean-inspired anti-inflammatory diet. CRP: C-reactive protein. CSS: Cross-sectional study. MED: Mediterranean diet. IBDQ: Inflammatory Bowel Disease Questionnaire. BMI: Body mass index. BW: Bodyweight. CC: ISO Country Codes. CD: Crohn’s Disease. EG/CG: Experimental and Control Group. FE: Serum iron. HDL: High-density lipoprotein. LDL: Low-density lipoprotein. TG: Triglycerides. GGT: γ-glutamyl transferase. LDH: Lactate dehydrogenase. SGOT: Glutamic-oxaloacetic transaminase. SGPT: Glutamic-pyruvic transaminase. ALP: Alkaline phosphatase. IL-6: Serum interleukin-6. IL-10: Serum interleukin-10. HBI: Harvey–Bradshaw Index. LFD: Low FODMAP diet. SIBDQ: Short IBD questionnaire. IBS-QOL: IBS quality of life questionnaire. IBS-SSS: IBS severity score system. BS: Bristol Stool. CIBSC: Copenhagen IBS disease courses. FARS: FODMAP adherence report scale. VAS: visual analogue scales. FODMAP: Fermentable oligo-, di-, and monosaccharides and polyols. ND: Normal diet. SCCAI: Simple clinical colitis index. FC: Fecal calprotectin. FFQ: Food frequency questionnaire. VHI: Van Hees index. IP: Intestinal permeability. LCLM: Low calcium low microparticles diet. LCNM: Low calcium normal microparticles diet. NCLM: Normal calcium low microparticles diet. NCNM: Normal calcium normal microparticles diet. HEMA: Hematocrit. CA: Plasma calcium. ALB: Albumin. DMF-UC: Dietary modification framework for UC. IBDQ-UK: United Kingdom version of Inflammatory Bowel Disease. PMS: Partial Mayo score. RB: Rectal bleeding. SF: Stool frequency. PGA: Physician global assessment. MS: Mayo score. MF: Mucosa friability. EIMs: Extraintestinal manifestations. TRF: Transferrin. PA: Prealbumin. GISD: Gastrointestinal symptom diary. KED: Komperod Elimination diet. GFD: Gluten-free diet. GFDQ: Gluten-free diet questionnaire. CCS: Case-control study. TAD: Typical Australian diet. FM: Fecal microbiota. FWC: Fecal water content. FSCFA: Fecal short-chain fatty acids. PDAI: Pouchitis disease activity index. SUR: Surgery. CS: Cohort study. FL: Fecal lactoferrin. DH: Dietary history. IEC: Initial episode cases. RC: Relapse Cases. PBD: Plant-based diet. PBDS: Plant-based diet score. ED: Exclusion diet. GSRS: Gastrointestinal symptom rating scale. 7-FR: 7-day food record. SRFGS: Satisfactory relief of functional-like gastrointestinal symptoms. RCS: Retrospective cohort study. RCD: Refined carbohydrate diet. SVD: Semi-vegetarian diet. VD: Vegetarian diet. DQ: Dietary questionnaire. MTWAI: Modified Truelove and Witts activity index. SF-36: Short Form-36 health survey. SCD: Specific Carbohydrate diet. IBS: Irritable bowel syndrome. CLD: Celiac disease. WBC: White blood cells. PL: Platelets. IGED: Inmunoglobulin Exclusion Diet. LMD: Low microparticles diet. NMD: Normal microparticles diet. AIPD: Autoimmune protocol diet. MCDAI: Modified CDAI. MED: Mediterranean Diet.

**Table 3:** Methodological quality analysis according to the CONSORT (Consolidated Standards of Reporting Trials) guide for reporting clinical trials.

Studies	1	2	3	4	5	6	7	8	9	10	11	12	13	14	15	16	17	18	19	20	21	22	23	24	25	Total Score	(%)
Berghouse et al., 1984 [38]	0	1	0	0.5	1	0.5	0	0	0	0	0.5	0.5	1	0.5	0	1	0.5	0	0	1	1	1	0	0	1	11/25	44
Jones et al., 1985 [39]	0	1	1	1	1	0.5	0	0	0	0	0	0	1	0.5	1	1	0.5	1	0	1	1	1	0	0	1	13.5/25	54
Ritchie et al., 1987 [40]	0	1	0.5	1	1	0.5	0.5	1	1	1	1	0.5	0.5	1	1	1	0.5	0	0	1	1	1	0	0	1	17/25	68
Lomer et al., 2001 [41]	1	1	0.5	0.5	1	0.5	0	0.5	0	0	1	1	1	0.5	1	1	0.5	0	0	1	1	1	0	0	0	14/25	56
Lomer et al., 2005 [42]	0.5	1	0.5	1	1	0.5	0	1	0	0	0.5	1	0.5	1	1	1	0.5	1	0	1	1	1	0	1	1	17/25	68
Bentz et al., 2010 [45]	0.5	1	0.5	0.5	1	0.5	0	1	0	0	1	0.5	1	0.5	0	0	0.5	0	0	1	1	1	0	0	1	12.5/25	50
Rajendran et al., 2010 [47]	0.5	1	0.5	0.5	1	0.5	0	NA	NA	NA	NA	0.5	NA	0.5	1	1	0.5	NA	0	1	1	1	NA	NA	NA	10.5/16	66
Uzunismail et al., 2011 [48]	0.5	1	0.5	0.5	1	0.5	0	NA	NA	NA	NA	0.5	1	0	1	0	0.5	NA	0	1	1	1	NA	NA	NA	10/17	59
Marlow et al., 2013 [49]	0.5	1	1	1	1	0.5	0	NA	NA	NA	NA	0	NA	0.5	0	1	0.5	NA	1	1	1	1	NA	NA	NA	11/16	69
Kyaw et al., 2014 [51]	1	1	0.5	1	1	0.5	0	1	0	1	0.5	1	1	0.5	1	1	1	0	0	1	1	1	0	0	1	17/25	68
Gunasekeera et al., 2016 [52]	1	1	0.5	1	1	0.5	0.5	1	1	1	1	1	1	0.5	1	1	1	1	0	1	1	1	0	0	1	20/25	80
Halmos et al., 2016 [53]	1	1	0.5	0.5	1	0.5	0.5	1	0	1	1	1	1	1	0	1	1	0	0	1	1	1	1	1	1	19/25	76
Komperod et al., 2017 [57]	1	1	0.5	1	1	0.5	0	NA	NA	NA	NA	0.5	NA	1	1	1	0.5	NA	0	1	1	1	NA	NA	NA	11.5/16	72
Konijeti et al., 2017 [58]	0.5	1	0.5	1	1	0.5	0	NA	NA	NA	NA	1	NA	0.5	1	1	1	NA	1	1	1	1	NA	NA	NA	13/16	81
Pedersen et al., 2017 [59]	1	1	0.5	1	1	0.5	1	0.5	0	1	0.5	1	1	0.5	1	1	1	1	0	1	1	1	0	0	1	18.5/25	74
Jian et al., 2018 [61]	0.5	1	0.5	1	1	0.5	0	1	1	0	0.5	0.5	1	1	1	1	1	0	0	1	1	1	0	0	1	16.5/25	66
Testa et al., 2018 [62]	0.5	1	0.5	1	1	0.5	0	NA	NA	NA	NA	0.5	0.5	1	1	1	0.5	NA	0	1	1	1	NA	NA	NA	12/17	71
Bodini et al., 2019 [63]	1	1	0.5	0.5	1	0.5	0	0.5	0	0	1	1	1	0.5	1	1	1	0	0	1	1	1	0	0	0	14.5/25	58
Cox et al., 2020 [67]	1	1	0.5	1	1	0.5	1	1	1	1	1	1	1	0.5	1	1	1	1	1	1	1	1	1	1	1	23.5/25	94

NA: Not applicable.

**Table 4:** Methodological quality analysis according to the STROBE (Strengthening the Reporting of Observational studies in Epidemiology) guide for reporting observational studies.

Studies	1	2	3	4	5	6	7	8	9	10	11	12	13	14	15	16	17	18	19	20	21	22	Total Score	(%)
Heaton et al., 1979 [37]	0.5	1	1	1	1	0.5	1	1	0	0	0	0.4	0.66	1	1	0.33	0	0	0	1	1	1	11/22	50
Croagh et al., 2007 [43]	0.5	1	1	1	0	0	1	1	0	0	0	0.4	0.66	1	1	0.33	1	1	1	1	1	1	13/22	59
Gearry et al., 2008 [44]	0.5	1	1	1	1	1	1	1	0	0	0	0.6	0.66	1	1	0.33	1	1	1	1	1	1	15/22	68
Chiba et al., 2010 [46]	0.5	1	1	1	1	0.5	1	1	0	0	0	0.6	0.66	1	1	0.33	1	1	1	1	1	0	13/22	59
Herfarth et al., 2014 [50]	0.5	1	1	1	1	0	1	1	0	0	1	0.6	0.33	0.5	1	0.33	0	1	1	1	1	1	13/22	59
Maagard et al., 2016 [54]	1	1	1	1	1	1	1	1	0	0	1	0.6	0.66	1	1	0.66	1	1	1	1	1	1	17/22	77
Prince et al., 2016 [55]	0.5	1	1	1	1	0.5	1	1	1	0	1	0.6	0.33	1	1	0.66	1	1	1	1	1	1	16/22	73
Suskind et al., 2016 [56]	0.5	1	1	1	1	0	1	1	0	0	1	0.2	0.33	1	1	0.33	0	1	1	1	1	1	14/22	64
Chiba et al., 2018 [60]	0.5	1	1	1	1	0.5	1	1	0	0	1	0.6	0.66	1	1	0.33	1	1	1	1	1	1	15/22	68
Godny et al., 2019 [64]	0.5	1	1	1	1	0.5	1	1	0	0	1	0.4	0.33	1	1	0.33	0	1	1	1	1	1	14/22	64
Papada et al., 2019 [65]	0.5	1	1	1	1	1	1	1	0	0	1	0.6	1	0.5	1	0.33	1	1	1	1	1	1	16/22	73
Schreiner et al., 2019 [66]	0.5	1	1	1	1	1	0	1	0	0	1	0.6	0.33	1	1	0.66	1	1	1	1	1	1	15/22	68

**Table 5:** Influence analysis in a meta-analysis using the leave-one-out method (random effect).

			Meta−Analysis for: Effect Size (%Heterogeneity)			
ID	Omitting	*n*	CDAI	FC	CRP	ALB
1	Lomer et al., 2001 [41]	10	−125 (93.5%)			
2	Lomer et al., 2001 [41]	10	−82 (89.5%)			
3	Lomer et al., 2005 [42]	41	−104 (96.7%)			
4	Lomer et al., 2005 [42]	42	−109 (96.7%)			
5	Chiba et al., 2010 [46]	22	−97 (96.6%)		0.1 (0.0%)	0.13 (67.4%)
6	Chiba et al., 2010 [46]	11	−121 (96.6%)		−1.6 (80.6%)	0.14 (67.4%)
7	Rajendran et al., 2010 [47]	29	−113 (96.7%)			
8	Marlow et al., 2013 [49]	8			−0.8 (85.0%)	
9	Halmos et al., 2016 [53]	8		−6.8 (32.3%)		
10	Konijeti et al., 2017 [58]	6		−5.1 (0.0%)	−1.0 (85.2%)	0.29 (0.0%)
11	Pedersen et al., 2017 [59]	37		−23.1 (29.5%)	−1.2 (83.2%)	
12	Komperod et al., 2017 [57]	6		−34.2 (14.0%)		
13	Jian et al., 2018 [61]	49				0.07 (44.5%)
	Pooled estimate		−107 (96.1%)	−7.4 (2.3%)	−0.9 (80.3%)	0.16 (51.7%)

CDAI: Crohn´s Disease Activity Index. FC: Fecal Calprotectin. CRP: C-Reactive Protein. ALB: Albumin.

## Data Availability

Not applicable.
